# A Critical Cross-Species Comparison of Pollen from *Nelumbo nucifera* Gaertn. vs. *Nymphaea lotus* L. for Authentication of Thai Medicinal Herbal Tea

**DOI:** 10.3390/plants9070921

**Published:** 2020-07-21

**Authors:** Duangjai Tungmunnithum, Sullivan Renouard, Samantha Drouet, Jean-Philippe Blondeau, Christophe Hano

**Affiliations:** 1 Department of Pharmaceutical Botany, Faculty of Pharmacy, Mahidol University, Bangkok 10400, Thailand; 2 Laboratoire de Biologie des Ligneux et des Grandes Cultures, INRAE USC1328, University of Orleans, 45067 Orléans CEDEX 2, France; samantha.drouet@univ-orleans.fr; 3 Bioactifs et Cosmetiques, CNRS GDR 3711, 45067 Orléans CEDEX 2, France; 4 Institut de Chimie et de Biologie des Membranes et des Nano-objets, CNRS UMR 5248, Bordeaux University, 33600 Pessac, France; sullivan.renouard@u-bordeaux.fr; 5 Conditions Extrêmes et Matériaux: Haute Température et Irradiation (CEMHTI) CNRS UPR3079, 1D Avenue de la Recherche Scientifique, 45071 Orléans, France; jean-philippe.blondeau@univ-orleans.fr

**Keywords:** authentication, flavonoids, FTIR, herbal medicine, lotus, *Nelumbo nucifera* gaertn., *Nymphaea lotus* L., pollen, scanning electron microscopy

## Abstract

“Bau Luang” or *Nelumbo nucifera* Gaertn. is an aquatic medicinal herb that has been used as a component of traditional medicines, medicinal products, and herbal tea for good health, particularly in Asia. The stamen of *N. nucifera* is an important part of this medicinal plant that is used in the form of dried and/or powdered stamens for herbal tea as well as the main ingredient of some traditional remedies. However, there is another aquatic herb called “Bau Sai” or *Nymphaea lotus* L. that is distributed in similar locations. Living plants of these two aquatic species may be classified according to their morphology, but the dried and powdered stamens of these two medicinal species are difficult to distinguish. The major reason of adulteration is the higher price of Bau Luang stamen. As a result, various methods of authentication, such as pollen micromorphology evaluation using scanning electron microscopy (SEM) analysis, bioinformatics analysis of two nuclear and plastic DNA markers, phytochemical stamen profiling, and Fourier transform infrared (FTIR) analysis of stamen plant material authentication from Bau Luang and Bau Sai, have been used in this present research in order to avoid some adulteration and/or misuse between the dried stamens of Bau Luang and Bau Sai. These results showed that the micro-morphology of pollen (size of pollen grain, number of apertures, and surface ornamentation) from the SEM analysis, some phytochemical compounds and the FTIR sporopollenin-to-protein ratio signal analysis are potential tools for authentication and identification of these two medicinal plants from their dried-stamen materials. This model of investigation may also be used to distinguish dried plant material from other problematic plant groups.

## 1. Introduction

The stamen of *Nelumbo nucifera* Gaertn. is one of the important ingredients of traditional medicines in Asian countries, e.g., China, Thailand, Japan, and India. It has also been used as an herbal tea for health benefits including boosting the body’s immune system and improving circulation. *N. nucifera* is an aquatic species member of the family Nelumbonaceae and is mainly distributed in Asia, especially in Thailand, Sri Lanka, India, China, and Nepal [1,2,3,4,5,6,7,8]. The dried or powdered stamen of *N. nucifera* stamen is often used to prepare traditional remedies and herbal tea [2,7,9].

However, the plants collected may be misidentified, contaminated, or falsified with other species. In Thailand, local people in many localities of the country collect and prepare the dried or powdered stamens of *N. nucifera* for sale as local products that can be accessed by both Thai people and foreign travelers. *N. nucifera* is also called “Bau Luang,” its vernacular name in Thailand. However, there is another aquatic medicinal plant that shares the similar vernacular name “Bau Sai” and has a similar distribution. Its scientific name is *Nymphaea lotus* L. The whole flower of “Bau Sai” has been commonly used for mild fever treatment, but the popular part used of this plant are the petiole and peduncle, which are used as a vegetable for cooking. According to the lack of research on the bioactive molecule and the pharmacological activity and safety of “Bau Sai,” there are no herbal teas made from this species in the market. Generally, these two aquatic medicinal plants can be distinguished by the morphological characteristics of living plants. Nonetheless, dried specimens of these medicinal herbs (particularly the stamens) are difficult to distinguish.

Either in academic research (e.g., prior to the evaluation of the biological activity of a medicinal plant) or in industry (e.g., prior to the placing on the market of a product that is expected to be bioactive and safe), the precise authentication of the starting raw plant material is the crucial starting point. First, research cannot be considered scientifically valid if the studied plant material has not been authenticated and characterized so that it can be replicated and produced on a larger scale. Second, for medicinal uses, a number of factors may affect the quality, efficiency, and safety of plant-derived products, and even subtle variations in raw materials may have dramatic effects on these three factors. Various authentication techniques can range from botanical or morphological plant identification to more complex approaches based on genetic and/or analytical chemistry methods [10]. While information obtained from morphological (scanning electron microscopy), genetic (DNA), and chemical (liquid chromatography coupled to mass spectrometry) composition analyses of pollen is useful for authentication purposes, these analyses can be complex and time-consuming. As a result, FITR analysis is sometimes preferred. In particular this method has shown great potential for quick and economical chemical characterization, identification, and pollen classification [11].

There are some adulterations or misused between Bau Luang and Bau Sai in herbal medicines or products made by the stamens of Bau Luang (*N. nucifera*). This is probably because the price of Bau Luang’s stamen is much more expensive than that of Bau Sai. Besides, local people and the foreigners are confident in the safety of the well-known medicinal plant, i.e., Bau Luang, as its stamens have long been used as herbal tea and various traditional remedies. Thus, authentication methods are needed to clearly authenticate dried plant material from these two medicinal plant species. This present study aims to evaluate different botanical, molecular, and chemical approaches to propose some effective tools for the authentication the dried plant material from these two species using their stamens.

## 2. Results and Discussion

### 2.1. Macro- and Micro-Morphological Evaluations as Tools for N. nucifera vs. N. lotus Authentication

**Figure 1:**
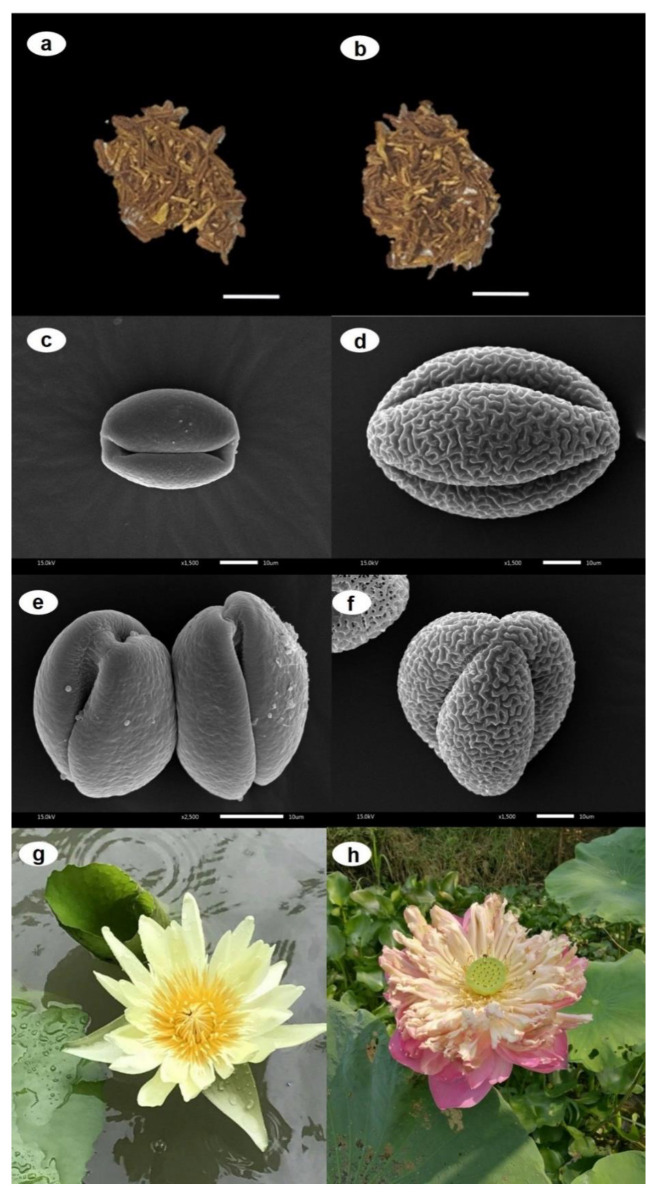
The dried stamens of (**a**) *Nymphaea lotus* (bar scale = 0.5 cm) and (**b**) *Nelumbo nucifera* (bar scale = 0.5 cm). The scanning electron microscopy (SEM) analysis of pollen from (**c**,**e**) *N. lotus* (bar scale = 10 µm) and (**d**,**f**) *N. nucifera* (bar scale = 10 µm). The living specimens of (**g**) *N. lotus* and (**h**) *N. nucifera* from the natural habitats. Photo by Duangjai Tungmunnithum.

*N. nucifera* and *N. lotus* share common macro-morphological characters such as simple leaves with long and slender petiole and a solitary flower with long peduncle. Their perianths vary in color, shape, and size (Figure 1).

The key characters from their macro-morphology of living plants that are helpful to discriminate between these two aquatic medicinal species are leaf surface, receptacle, ovary, number of carpels, and placentation, as shown in Table 1.

These key characteristics can be easily observed in the living plant (Figure 1g,h) but are difficult to observed in the dried plants. The dried stamens of *N. nucifera*, which have long been used to prepare the herbal medicines and/or herbal tea products, are quite similar to those of *N. lotus* (Figure 1a,b).

Interestingly, micro-morphological evaluation from the present study indicates that pollen micro-morphology is an effective tool to authenticate the dried stamens in herbal tea and herbal medicines. This is the first report on the potential of dried stamen for the authentication of raw plant material of *N. nucifera.* The scanning electron microscopy (SEM) analysis of *N. nucifera* and *N. lotus* pollens illustrated that the pollen grain of both species is furrow-shaped. The sizes (mean ± S.D.) of the polar and equatorial axes (P/E ratio) of *N. nucifera* and *N. lotus* pollens is 34.26 ± 0.71 and 30.57 ± 2.37 µm, respectively. The pollen grain of *N. lotus* is smaller than that of *N. nucifera* (Figure 1c,d). In addition, *N. lotus* contains 2-colporate pollen grains (Figure 1e), while *N. nucifera* consists of 3-colporate pollen grains (Figure 1f). The surface ornamentation of pollen grain of both aquatic herbs is also clearly different. The pollen surface of *N. lotus* has verrucated ornamentation with microgranules, whereas that of *N. nucifera* has regulated ornamentation without microgranules (Figure 1c,d). The pollen micro-morphology evaluation using SEM analysis was also recorded as a useful tool to identify other plant groups, i.e., *Hoya siamica* complex species [12]; *Betula utilis*, *B. maximowicziana*, *B. dahurica*, *B. pubescens*, *B. pendula*, and *B. humilis* [13]; species members of Mimosaceae [14]; species members of Papilionaceae [15]; etc.

### 2.2. Bioinformatic Analysis of Two Nuclear and Plastid DNA Markers as a Tool for the Authentication of N. nucifera vs. N. lotus

**Figure 2:**
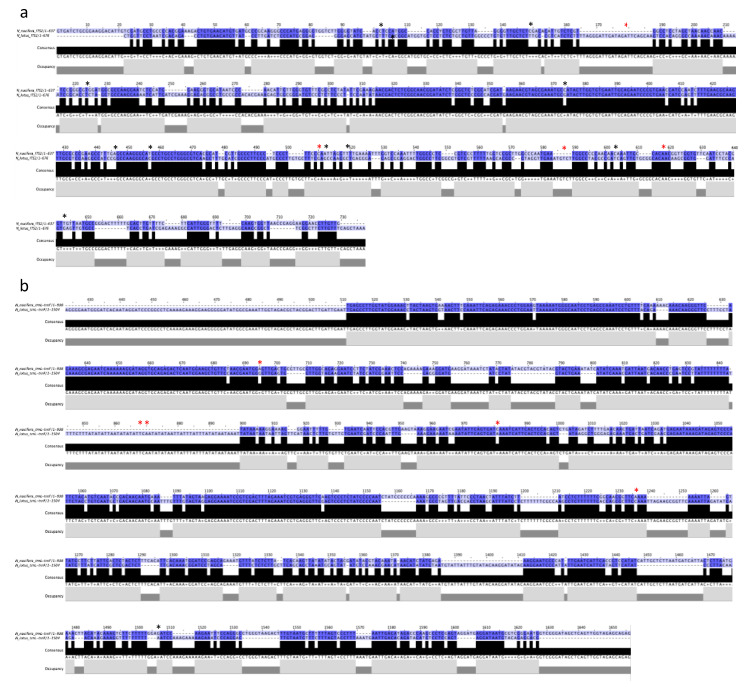
DNA sequence alignments showing consensus and occupancy for the nuclear internal transcribed spacer 1 (*ITS1*) (**a**) and the chloroplast tRNA-Leu-tRNA-Phe (*trn*L-*trn*F) intergenic spacer (**b**) from *N. nucifera* vs. *N. lotus*. Intraspecific variable positions are indicated by * (red) for *N. nucifera* and * (black) for *N. lotus*.

To evaluate the possible use of DNA markers to discriminate these two lotus species, a search for DNA sequences including the nuclear internal transcribed spacer 1 (*ITS1*) and internal transcribed spacer 2 as well as the chloroplast *tRNA-Leu-tRNA-Phe* (*trn*L-*trn*F) intergenic spacer was performed on the GenBank database for both *N. nucifera* and *N. lotus*. Our search retrieved 20 and eight *ITS1* sequences, as well as three and eight *trn*L-*trn*F sequences, for *N. nucifera* and *N. lotus*, respectively. Retrieved sequences lengths ranged from 626 bp to 737 bp and from 280 bp to 677 bp for *ITS1* and from 998 bp to 2059 bp and from 1503 bp and 1520 bp for *trn*L-*trn*F for *N. nucifera* and *N. lotus*, respectively. All positions that contain gaps and missing data were removed for sequence alignments. After sequence alignment, canonical sequences for each DNA sequence were obtained—637 bp and 676 bp for *ITS1*, and 998 bp and 1504 bp for *trn*L-*trn*F, respectively, for *N. nucifera* and *N. lotus* (Figure 2). Hypervariable regions were identified between the sequences of these two species (Figure 2). However, some of these nucleotide variations correspond to the intra-specific variations identified during alignment, performed in order to obtain canonical sequences, as shown in Figure 2.

As a first step in the possible use of DNA barcoding to distinguish *N. nucifera* from *N. lotus* stamens, we assessed the suitability of two conventionally used DNA sequences. DNA barcoding is now widely used to identify and classify plant species using short DNA sequences from nuclear or intracellular organelles. This technique was first proposed for animal species using DNA sequence coding for the mitochondrial enzyme cytochrome oxidase 1 (CO-1) [16]. In plants, mitochondrial genes are unsuited for generating DNA barcodes due to their slow evolution at very low substitution rates. The alternative is the use of nuclear and chloroplast genomes, which have much higher substitution rates. In particular, many regions of the chloroplast genome have been assessed for their possible use in plant DNA barcoding [17]. *Nymphaea* is the richest *Nymphaeales* genus in number of species and is the most phenotypically diverse and geographically widespread (nearly worldwide). In plant systematics, the chloroplast region including the intergenic spacer *trn*L-*trn*F is among the most commonly used non-coding DNA regions [18]. Phylogenetic separation of 35 *Nymphaea* species analyzed (out of the estimated 45–50 species) was possible based on a single analysis of the *trn*T-*trn*F chloroplast region (including *trn*L) [19]. This chloroplast *trn*L-*trn*F region has also been shown to be essential for studying the molecular evolution of land plants [18]. In particular, *N. nucifera*, considered a key species as a late Cretaceous relic, is classically included in these molecular evolution studies [18], while *N. lotus* is considered an emerging model for this Darwin’s abominable mystery (i.e., the birth and rapid radiation of flowering plants) [20]. Recent works have also demonstrated the improved ability to identify closely related species by combining the internal transcribed spacer 1 (*ITS1*) nuclear DNA sequence to this chloroplast DNA sequence [17]. Although DNA-based technologies in the authentication process are clearly superior to other approaches, it should be noted that they have a number of drawbacks in that they need high-quality DNA, which is not always compatible with the treatments used in the processing, drying, or storage of raw materials. Many markers may exhibit intraspecific sequence variation, which compromises their use for DNA barcoding approaches. Here, the sequence comparison of these two nuclear and chloroplast DNA sequences of *N. nucifera* and *N. lotus* revealed hypervariable regions between the two species and thus clearly demonstrated that the use of these two sequences to discriminate these two species could be envisaged (Figure 2). Several intraspecific variations in the nucleotide sequences of these two universally used markers have also been observed for each species (Figure 2). It is therefore important to take these intra-specific variations into account and not to regard these nucleotide positions as discriminatory for the authentication of the two species. Such intraspecific variations have already been reported in other plant species and could constitute limits for the use of DNA barcoding in plant authentication [10,21].

### 2.3. Flavonoids Profiling in Stamen Extracts as a Tool for the Authentication of N. nucifera vs. N. lotus

**Figure 3:**
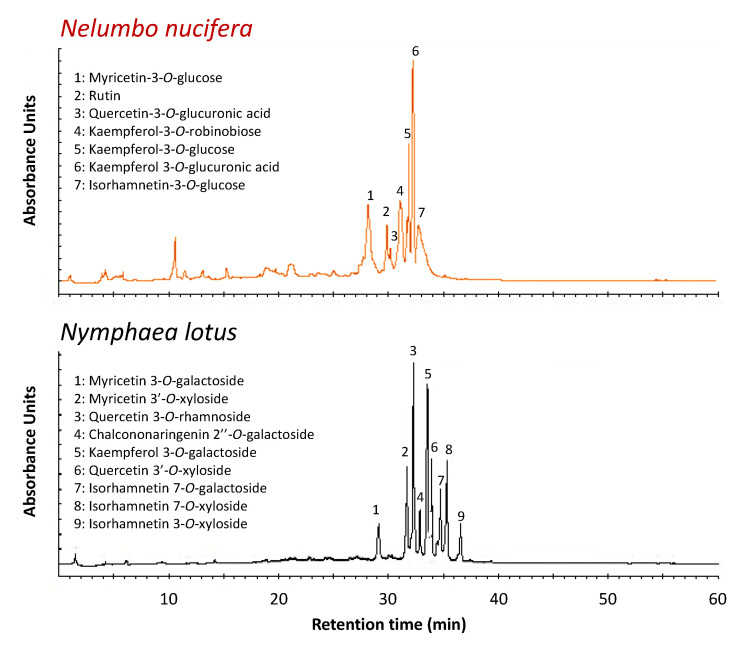
Representative high-performance liquid chromatography (HPLC) chromatograms of extracts from stamens of *N. nucifera* and *N. lotus*.

Flavonoids were successfully used to interpret evolutionary relationships in a number of angiosperm groups as well as for botanical authentication [22,23]. Both *N. nucifera* [9,24,25,26] and *N. lotus* [27,28,29] were reported to accumulate large amounts of a wide variety of different flavonoids, and some of them were proposed as chemotaxonomic markers that might be used for authentication purposes. A representative high-performance liquid chromatography (HPLC) reference pattern was established for each species using an optimized extraction method designed for flavonoid extraction from lotus stamens [29]. These representative HPLC chromatograms are shown in Figure 3. Note that these reference patterns are representative of eighteen populations of *N. nucifera* and 13 populations of *N. lotus* collected throughout the different floristic Regions of Thailand. Only quantitative but not qualitative (i.e., different composition) variations have been identified (data not shown).

**Figure 4:**
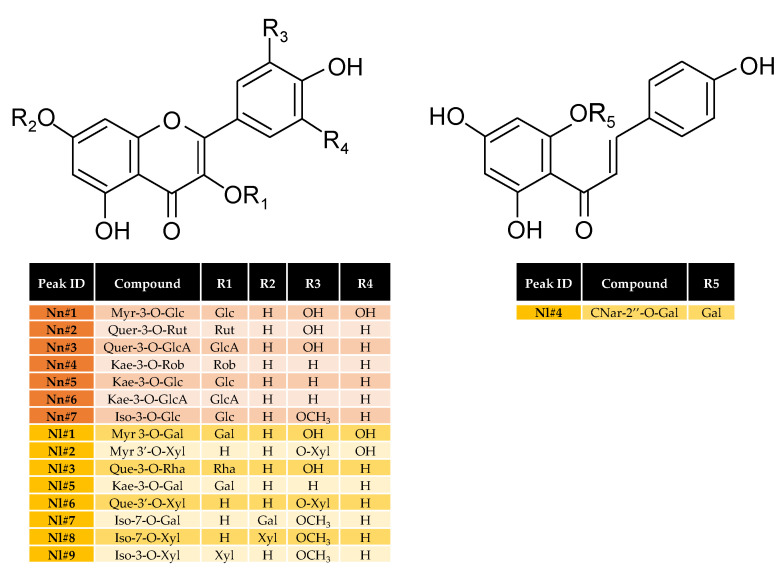
Structures of the mains flavonoids from *N. nucifera* and *N. lotus* stamen extracts. Myr: myricetin; Que: quercetin; CNar: chalcononaringenin; Kae: kaempferol; Iso: isorhamnetin; Rut: rutinose; Rob: robinobiose; GlcA: glucuronic acid; Glc: glucoside; Gal: galactoside; Xyl: xyloside; Rha: rhamnoside.

Based on LC-MS analysis, comparison with authentic standards, and literature data [25,27,28], seven major flavonoids were identified in stamen extract from *N. nucifera*, while nine major flavonoids were identified in stamen extract from *N. lotus* (Table 2; Figure 4).

*N. nucifera* was shown to accumulate only flavonol *O*-glycosides (FOGs), whereas *N. lotus* also accumulated one chalcone glycoside in addition to FOGs (Figure 3, Table 2). For both species, these flavonols were derived from only four different genins—quercetin (Que), kaempferol (Kae), myricetin (Myr), and isorhamnetin (Iso). However, the corresponding glycoside forms detected in the extracts were all specific to one species (Table 2).

Of the seven FOGs detected in *N. nucifera* stamen extract, three were derived from Kae (Kae-3-*O*-Rob, Kae-3-*O*-Glc, and Kae-3-*O*-GlcA), two from Que (Quer-3-*O*-Rut and Quer-3-*O*-GlcA), one from Myr (Myr-3-*O*-Glc), and one from Iso (Iso-3-*O*-Glc), with a preferential accumulation of Kae derivatives. Remarkably, *O*-glycosylation occurred at the hydroxyl group in position 3 of ring C for all of these flavonols. In the case of *N. lotus* stamen extract, three of the eight flavonol glycosides were derived from Iso (Iso-7-*O*-Gal, Iso-7-*O*-Xyl and Iso-3-*O*-Xyl), two from Myr (Myr 3-*O*-Gal and Myr 3′-*O*-Xyl), two from Que (Que-3-*O*-Rha and Que-3′-*O*-Xyl), and one from Kae (Kae-3-*O*-Gal). The glycosylation occurred at different positions from the three rings. In contrast to *N. nucifera*, a preferential accumulation of glycosides derived from one genin was not observed for *N. lotus*. In addition, the presence of one chalcone glycoside derived from chalcononaringenin (CNar-2″-*O*-Gal) was detected. The relative quantification of each flavonoid glucosides from *N. lotus* and *N. nucifera* stamen extracts is presented in Table S1.

Flavonoids are among the most prevalent group of plant secondary metabolites. More than 5000 naturally occurring flavonoids have been described to date. They have been classified according to their chemical structure, usually subdivided into different subgroups that can be accumulated differently depending on the species. Due to this diversity, some flavonoids have been successfully used to interpret evolutionary relationships and for botanical authentication of angiosperms. Specific accumulation of some flavonoid *C*-glycosides (FCGs) has been proposed for authentication as possible chemotaxonomic markers [30]. The presence of FCGs was reported in *N. nucifera*, but their accumulation was mainly restricted to the embryo [9,24,25,26], and, in good agreement with our present analysis, FCGs were not previously detected in stamens [25]. In view of the wide distribution of FOGs in the plant kingdom, and although these FOGs are all different in the present extracts, it would not be reasonable to consider their use for the authentication of the two lotus species. By contrast, the chalcones have already shown their usefulness in establishing evolutionary and systematic connections between plants [22], so we propose that the chalcononaringenin derivative detected here in the *N. lotus* (absent in *N. nucifera*) could be the preferred authentication marker.

However, to reach the maximum potential of these compounds, the influence of the cultivars, developmental stages, parts of plant, seasons, and time to harvest should be considered. Here, as qualitative variations (with totally different flavonoids are accumulated between the two species) are observed, it is anticipated that at least some of the identified compounds could serve as chemical markers to discriminate between the two species.

### 2.4. Pollen FTIR Analysis as a Tool for the Authentication of N. nucifera vs. N. lotus

**Figure 5:**
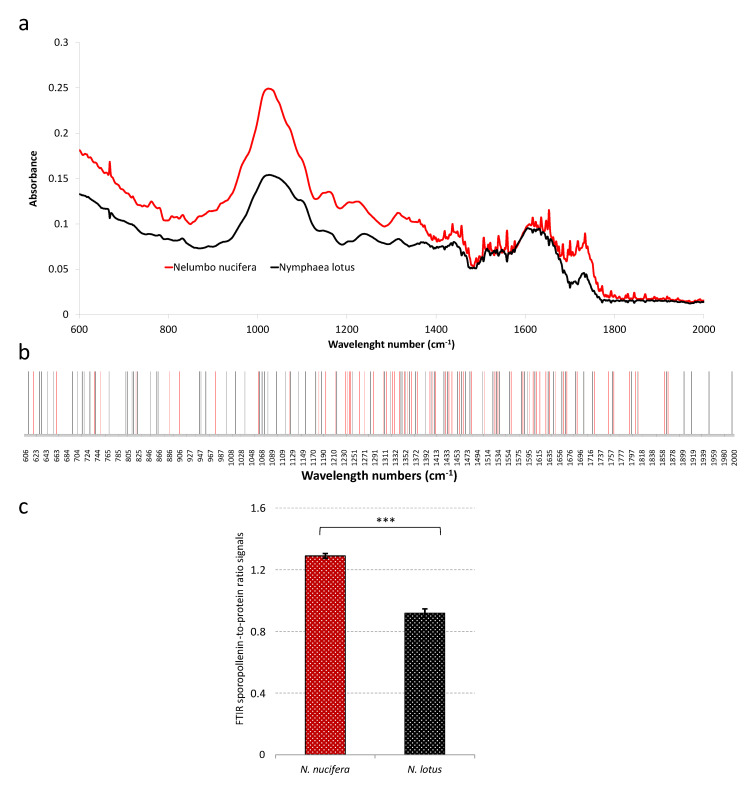
Typical Fourier transform infrared (FTIR) spectra of pollen from *N. nucifera* and *N. lotus* (**a**); specific bands possibly used for *N. nucifera* and *N. lotus* pollen authentication (**b**); FTIR sporopollenin-to-protein ratio signals (at 883 cm^−1^ and 1659 cm^−1^, respectively) for *N. nucifera* and *N. lotus* pollen authentication (**c**). *** significant *p* < 0.001.

Fourier transform infrared (FTIR) spectroscopy is a non-destructive method that allows a simple and quick analysis of the chemical and physical components of the sample. It was widely used for sample authentication in the food, pharmaceutical, and cosmetic industries [10,31]. FTIR can discriminate between species highlighting IR bands present in only one species (qualitative markers) or IR bands having a significant difference between species (quantitative markers). This technique also provided important information for the identification of pollen [11,13]. Figure 5a shows the FTIR spectra of the *N. nucifera* and *N. lotus* pollen materials.

To avoid any problems generally encountered with biological samples (especially the possible presence of different quantities of water in the sample) [32], to propose a reproducible analysis, and in good agreement with previous study with pollen [11], we focused our analysis on the spectrum region 2000–600 cm^−1^. In this analyzed region, some statistically significant specific bands possibly used for *N. nucifera* and *N. lotus* pollen authentication are also proposed (Figure 5b). Most of these specific bands are in the region of 1700–800 cm^−1^. In particular, these wave number could originate from the chemical bonds of polysaccharides (around 1200–900 cm^−1^ (C−O, C−C, C−O−C, and C−OH stretches and deformations)), proteins (around 1650–1700 (amide I), 1535 (amide II), and 1445 cm^−1^ (CH_2_ deformation)), lipids (around 1745 (C=O stretch), 1460 (CH_2_ deformation) and 1165 cm^−1^ (C−O−C stretching in esters)), and polyamines (around 1605, 1515, 1205, 1170, 855, 830 and 815 cm^−1^) [11,13,33]. Proteins, acyl-lipids, polysaccharides, sporopollenin conjugates composed mainly long chain fatty acids, and diverse phenylpropanoids including hydroxycinnamic acids were all reported in different concentrations on the surface of the cell walls of mature pollen of all higher plants [11,34]. Here, a majority of the *N. nucifera*-specific bands were located in the 1200–800 cm^−1^ region, while an important part of the *N. lotus*-specific bands was observed in the 1700–1500 cm^−1^ region of the FTIR spectra. This may be due to the differential accumulation of these components of the pollen cell wall in the two species with potentially higher sporopollenin accumulation for *N. nucifera* pollen and/or higher *N. lotus* protein content. This could be related to the difference in exine, the outer wall of pollen grain, ornamentation observed between the *N. nucifera* and the *N. lotus* (Figure 1). Exine is a sporopollenin-rich tissue [11]. FTIR ratio signals have been previously used; in particular, the protein-to-polysaccharide ratio signals have been proposed as taxon-specific [11]. Here, the sporopollenin-to-protein ratio signals (at 883 and 1659 cm^−1^, respectively) showed a significant difference between *N. nucifera* and *N. lotus* (Figure 5c). We proposed that the non-destructive, simple and quick analysis using FTIR sporopollenin-to-protein ratio signals could be used as an *N. nucifera* and *N. lotus* authentication tool. Future investigations will be carried out to further assess this possibility—in particular to assess seasonal, geographical, and/or inter-individual variations from a wide range of different populations.

## 3. Materials and Methods

### 3.1. Plant Materials

A living specimen of *N. nucifera* was searched for and collected from Sukhothai province, and that of *N. lotus* was collected from Nakhon Sawan province. Both of these localities (provinces) are located in the Northern Floristic Regions of Thailand. The living specimens of *N. nucifera* and *N. lotus* were then identified using the key-to-species and existing floras and compared with the herbarium specimens kept in BKF (Forest Herbarium) and BCU (the Professor Kasin Suvatabandhu Herbarium, Chulalongkorn University). Herbarium abbreviations are used according to Thiers [35]. Then, their stamens were cut from the flowers, and air-dried stamen sample were prepared following the World Health Organization, 1998 [36] for further experiments.

### 3.2. Macro- and Micro-Morphological Evaluations as Tools for N. nucifera vs. N. lotus

Macro-morphological characters of the whole plants belonging to *N. nucifera* and *N. lotus* were carefully examined for both vegetative and reproductive structures of collected living specimens from their natural habitats. The morphological characters were examined under both compound light microscope (LM) and dissecting stereo microscopes. Each character was measured three times per specimen, and their average was used. Linear measurements of macroscopic morphological characteristics were measured by using standard ruler or digital Vernier caliper. Micro-morphological characteristics were performed under a light microscope equipped with 10X lens coupled to a micrometer disc and 10X or 40X or 100X objectives. Then, the key characteristics that are helpful to identify these medicinal plant species were investigated.

For the micro-morphological characteristics of pollen, the pollen grains from the dried mature stamens of *N. nucifera* and *N. lotus* were prepared on the glass slides and observed at different magnifications using compound light microscope. Then, the scanning electron microscope (SEM) was prepared. The pollen samples *N. nucifera* and *N. lotus* were examined by SEM. Dry pollens without critical-point-dried process from each sample were placed on an aluminum stub and sputter-coated with gold for 5 min, then the pollen specimens were observed using SEM model JEOL JSM-5410 LV. The SEM micrographs were taken with 1000 to 15,000 magnification at 15 kV. The pollen terminology is according to Hesse et al. [37].

### 3.3. Bioinformatic Analysis

Searches for the nuclear internal transcribed spacer 1 (*ITS1*) and internal transcribed spacer 2 and the chloroplast *tRNA-Leu-tRNA-Phe* (*trn*L-*trn*F) intergenic spacer on the GenBank database after verification gave 20 (DQ105985, DQ105984, DQ105983, DQ105982, DQ105981, AF136290, AF136289, FJ599761, JF977133, JF977132, JF977131, JF977130, AY615194, JN407494, DQ901015, EF211986, EF211985, EF211984, AY858640, AY620418) and 8 (MK452746, MK452745, MK452744, MK452742, EU428063, MK452743, EU191034, FM242153) *ITS1* sequences, and 3 (FJ626571,AM397161, FJ626571) and 8 (MK452761, MK452760, MK452759, MK452757, AM422042, AM422041, AM422040, HF968682) *trn*L-*trn*F sequences, respectively, for *N. nucifera* and *N. lotus*. Sequence alignments were conducted in MEGA6 to determined canonical sequences. Intra-specific nucleotide variations were identified during this alignment. Then, alignments of canonical sequences were conducted with MUSCLE and formatted with Jalview 2.8 to determine consensus and occupancy.

### 3.4. Chemicals

For extraction and LC analysis, analytical grade or the highest available purity solvents were used (Merck Millipore, Saint-Quentin Fallavier, France). Deionized water was prepared with the help of the Milli-Q water purification system (Merck Millipore, Saint-Quentin Fallavier, France). Commercial standards were purchased from Extrasynthese (Genay, France) and Sigma-Aldrich (Saint-Quentin Fallavier, France).

### 3.5. Flavonoids Extraction

A stamen sample (100 mg), placed in 5 mL quartz tubes equipped with a vapor condenser, was extracted by ultrasound-assisted extraction in 1 mL 90% (*v*/*v*) aqEtOH in USC1200TH ultrasonic bath (Prolabo, Fontenay-sous-Bois, France) using optimized extraction conditions of 30 kHz frequency for 45 min at 45 °C [29]. The extract was then centrifuged 15 min at 5000× *g* (Heraeus Biofuge Stratos, Thermo Scientific, Illkirch, France), and the obtained supernatant was filtered through 0.45 μm of nylon syringe membranes (Merck Millipore, Saint-Quentin Fallavier, France). Flavonoid enrichment was obtained through an additional DAX-8 (Merck Millipore, Saint-Quentin Fallavier, France) macroporous resin purification step as previously described [29].

### 3.6. LC-MS Analysis

Analysis of the LC-MS was performed as described by Drouet et al. [38], with a Water 2695 Alliance coupled with a single quadrupole mass spectrometer ZQ (Waters-Micromass, Manchester, UK). LC-ESI-MS. MassLynx 4.0 software (Waters-Micromass, Manchester, UK) was used to acquire and process the data. The separation was obtained with a linear gradient—from a mix of 10:90 (*v*/*v*) to 100:0 (*v*/*v*) of solvent A (methanol) and solvent B (water + 0.05% (*v*/*v*) formic acid) at a flow rate of 1 mL/min during 60 min. The detection was set at 350 nm.

### 3.7. FTIR Analysis

Reflectance spectra were acquired using a Brucker (Palaiseau, France) V70 interferometer working under a dehydrated air flow in reflectivity mode, with an ATR accessory containing a gold crystal. The measurements were performed for wavenumbers situated in the middle of the infrared range (between 600 cm^−1^ and 2000 cm^−1^). The instrumental resolution was about 2 cm^−1^, and measurements were averaged on 32 scans. IR bands localization was determined using second derivative. IR bands was defined as specific when it is only met in one specie (taking into account the instrumental resolution, i.e., plus or minus 2 cm^−1^).

### 3.8. Statistical Analysis

Statistical analyses were carried out using XLSTAT 2019 (Addinsoft, Paris, France). Data composed of at least three independent replicates were presented using the means and standard deviations. Student *t*-test was carried out for statistical comparative analysis. Significant thresholds were considered at *p* < 0.05 (*), 0.01 (**), and 0.001 (***).

## 4. Conclusions

To summarize, the dried plant materials of Bau Luang (*N. nucifera*) and Bau Sai (*N. lotus*) can be clearly identified by micro-morphological pollen characters from the pollen SEM analysis, as well as the chalcononaringenin derivative detected by phytochemical stamen profiling. In addition, a non-destructive, simple, and rapid analysis such as FTIR sporopollenin-to-protein ratio signals is a new authenticated tool for distinguishing these two aquatic medicinal species was also proposed in this work. This current work is an example to emphasize that a living plant and a dried or powdered plant material may require a variety of authentication tools to provide the right raw material for the preparation of herbal tea and/or traditional medicines. This model of authentication methods may also be employed to distinguish other problematic plant groups. Moreover, the safety and pharmacological activities of Bau Sai are future works that may provide an additional potential raw plant material for the medical and pharmaceutical sector.

## Figures and Tables

**Table 1:** The key characteristics that can be easily observed from morphology of living specimens and helpful for species identification between *N. nucifera* vs. *N. lotus* living plants.

Key Morphological Characters	*N. nucifera* *	*N. lotus* *
Leaf surface	pubescent	glabrous
Receptacle	torus present	torus absent
Ovary	superior	half inferior
Number of carpels	1	many
Placentation	marginal	pareital

* The 30 samples from each locality (population) were studied in this section.

**Table 2:** Characteristic and probable identification of flavonoids from *N. nucifera* and *N. lotus* stamen extract.

Peak ID	Retention Time (min)	λmax (nm)	[M-H]^−^	Propable Identification	Commercial Standard	Reference
Nn#1	27.89	275, 354	479	Myr-3-O-Glc	+(ES)	[25]
Nn#2	29.79	257, 354	609	Quer-3-O-Rut	+(SA)	[25]
Nn#3	30.11	257, 353	477	Quer-3-O-GlcA	+(SA)	[25]
Nn#4	30.23	266, 346	593	Kae-3-O-Rob	+(SA)	[25]
Nn#5	30.73	266, 347	447	Kae-3-O-Glc	+(SA)	[25]
Nn#6	31.93	269, 345	461	Kae-3-O-GlcA	+(SA)	[25]
Nn#7	32.34	255, 356	477	Iso-3-O-Glc	+(ES)	[25]
Nl#1	29.11	263, 349	479	Myr 3-O-Gal	+(ES)	[27]
Nl#2	31.67	254, 305, 366	449	Myr 3′-O-Xyl	+(ES)	[27]
Nl#3	32.26	257, 348	447	Que-3-O-Rha	+(SA)	[27,28]
Nl#4	32.87	250, 366	433	CNar-2′’-O-Gal	-	[27,28]
Nl#5	33.52	265, 343	447	Kae-3-O-Gal	+(SA)	[27]
Nl#6	33.91	254, 366	433	Que-3′-O-Xyl	+(SA)	[27]
Nl#7	34.71	268, 350	477	Iso-7-O-Gal	-	[27,28]
Nl#8	35.51	252, 268, 352	447	Iso-7-O-Xyl	-	[27,28]
Nl#9	36.57	265, 343	447	Iso-3-O-Xyl	-	[28]

Myr: myricetin; Que: quercetin; CNar: chalcononaringenin; Kae: kaempferol; Iso: isorhamnetin; Rut: rutinose; Rob: robinobiose; GlcA: glucuronic acid; Glc: glucoside; Gal: galactoside; Xyl: xyloside; Rha: rhamnoside. Standards: + = available; - = not available; commercial standard purchased from ES (Extrasynthese, Genay, France) or SA (Sigma-Aldrich, Saint-Quentin Fallavier, France).

## Supplementary Materials

The following are available online at https://www.mdpi.com/2223-7747/9/7/921/s1. Table S1. Relative quantification (expressed in absorbance unit per g dry weight (DW)) of the different flavonoid glucosides in *N. lotus* and *N. nucifera* stamen extracts.
