# Supplementary material for: A Critical Cross-Species Comparison of Pollen from Nelumbo nucifera Gaertn. vs. Nymphaea lotus L. for Authentication of Thai Medicinal Herbal Tea
Source: Plants (Basel). 2020 Jul 21;9(7):921. doi: 10.3390/plants9070921 (PMC7412456; doi:10.3390/plants9070921)
Supplement: Supplementary file 1 [file plants-09-00921-s001.pdf]

Table S1. Relative quantification (expressed in absorbance unit per g dry weight (DW)) of the different flavonoid glucosides in the *N. lotus* and *N. nucifera* stamen extracts.

Compound	*N. lotus*	*N. nucifera*
**Myr 3-O-Gal**	76.9 ± 3.8	-
**Myr 3′-O-Xyl**	129.3 ± 6.7	-
**Que-3-O-Rha**	244.2 ± 5.1	-
**CNar-2″-O-Gal**	64.7 ± 3.6	-
**Kae-3-O-Gal**	200.2 ± 5.4	-
**Que-3′-O-Xyl**	117.2 ± 2.4	-
**Iso-7-O-Gal**	90.4 ± 3.3	-
**Iso-7-O-Xyl**	144.2 ± 5.7	-
**Iso-3-O-Xyl**	56.6 ± 1.7	-
[redacted]		
**Myr-3-O-Glc**	-	204.3 ± 10.0
**Quer-3-O-Rut**	-	13.5 ± 2.8
**Quer-3-O-GlcA**	-	55.2 ± 3.3
**Kae-3-O-Rob**	-	243.2 ± 11.5
**Kae-3-O-Glc**	-	325.8 ± 16.7
**Kae-3-O-GlcA**	-	564.7 ± 18.8
**Iso-3-O-Glc**	-	157.2 ± 10.7
